# Supplementary material for: Genome-Wide Characterization and Evolutionary Expansion of Poplar NAC Transcription Factors and Their Tissue-Specific Expression Profiles under Drought
Source: Int J Mol Sci. 2022 Dec 23;24(1):253. doi: 10.3390/ijms24010253 (PMC9820422; doi:10.3390/ijms24010253)
Supplement: Supplementary file 1 [file ijms-24-00253-s001.zip › captions.pdf]

Figure S1: Electrophoretic diagram of PCR products of PdNAC genes. Electrophoretic diagram of PCR clone product of *PdNAC013, PdNAC105, PdNAC055, PdNAC028, PdNAC021, PdNAC078, PdNAC095, PdNAC027, PdNAC049* and *PdNAC0086*, respectively;

Table S1: Physicochemical properties of the *PtrNAC* members;

Table S2: Ka/Ks of the *PtrNAC* members;

Table S3: *Cis*-element of the *PtrNAC* members;

Table S4: Subcellular localization prediction of the *PtrNAC* members;

Table S5: PCR Primers of the *PtrNAC* members;

Table S6: Real-time quantitative primers of the *PdNAC* members.
