# Supplementary figures and images for: Genome-Wide Characterization and Evolutionary Expansion of Poplar NAC Transcription Factors and Their Tissue-Specific Expression Profiles under Drought
Source: Int J Mol Sci. 2022 Dec 23;24(1):253. doi: 10.3390/ijms24010253 (PMC9820422; doi:10.3390/ijms24010253)

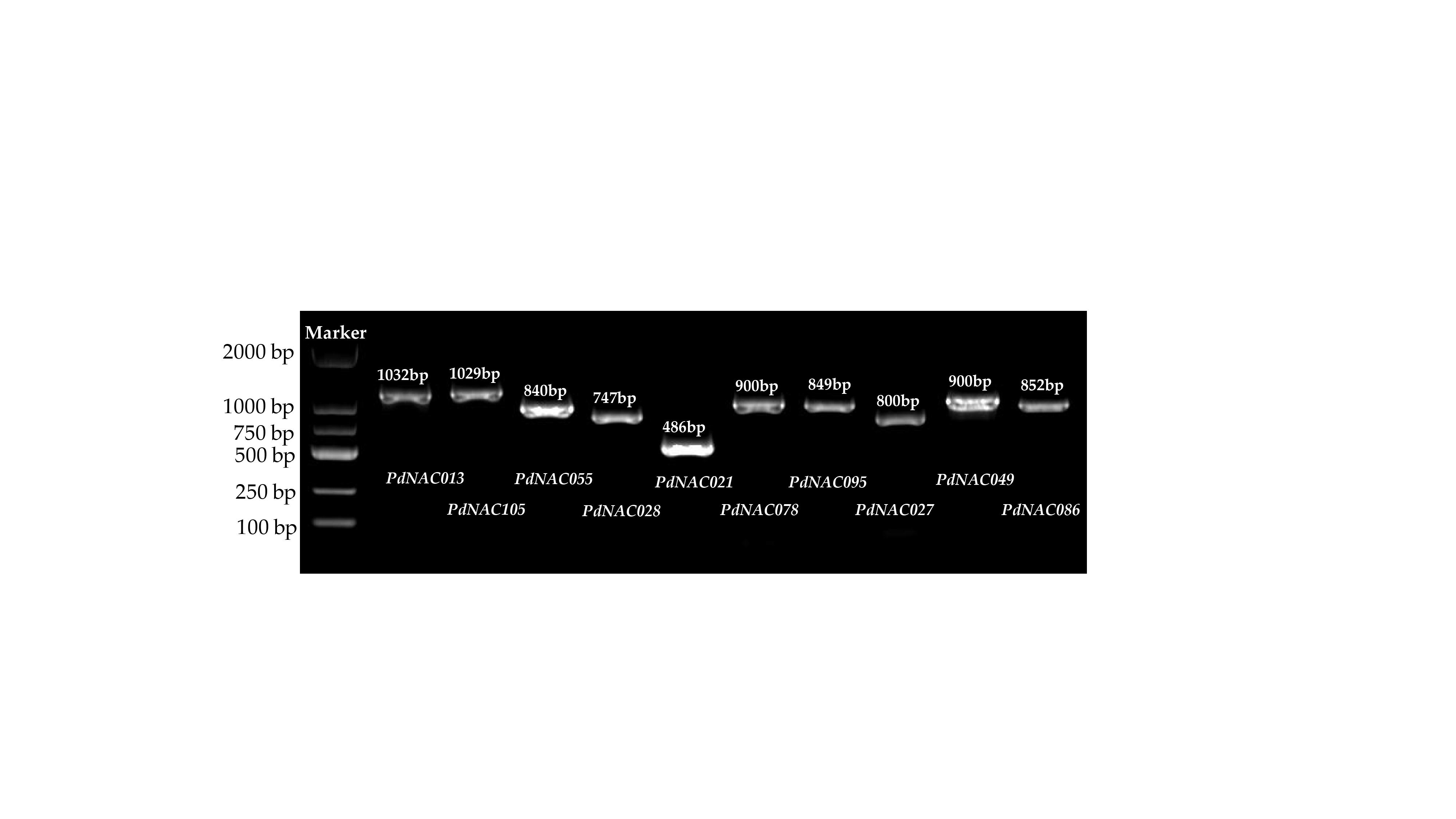

Supplement: Supplementary file 1 [file ijms-24-00253-s001.zip › Figure S1.jpg]
